# The Dental Laboratory

**Published:** 1890-12

**Authors:** 


THE DENTAL LABORATORY.—A Manual of Gold and Silver Plate Work for Dental Substitutes, Crowns, etc., Regulating Appliances, Repairing, etc., with manipulations in Vulcanite and Celluloid, Laboratory Hints, Suggestions, Fixtures, etc. By Theodore F. Chupein, D. D. S. Publishers: Johnson and Lund, Philadelphia, 1890.

This excellent treatise is dedicated to the Students of Dentistry attending the various Colleges in the United States and to those contemplating the study of dentistry. The work is profusely illustrated, and in the 120 pages, of which it consists, contains a mass of useful information, the result of actual experience on the part of its author, who is well known by a series of articles which have appeared in the *Dental Office and Laboratory*, of which he is the editor. It has been the aim of the author in preparing the work to treat and select all such cases as occur in the every-day practice of the dentist, both in the construction of new work and the repair of old, and to present there in the simplest and most explanatory manner, and his efforts to this end have certainly been successful, as a perusal of the work will show.

